# Both the “What” and “Why” of Youth Sports Participation Matter; a Conditional Process Analysis

**DOI:** 10.3389/fpsyg.2017.00659

**Published:** 2017-04-26

**Authors:** Siv Gjesdal, Paul R. Appleton, Yngvar Ommundsen

**Affiliations:** ^1^Department of Coaching and Psychology, Norwegian School of Sport SciencesOslo, Norway; ^2^School of Sport, Exercise and Rehabilitation Sciences, University of BirminghamBirmingham, UK

**Keywords:** youth sport, motivational regulation, goal orientation, self-esteem, competence, conditional process analysis

## Abstract

This study builds on previous research combining achievement goal orientation from Achievement Goal Theory and motivational regulation from Self-Determination Theory. The aim was to assess the combination of the “what” and “why” of youth sport activity, and how it relates to the need for competence and self-esteem. Achievement goal orientation, specifically task and ego, was employed to represent the “what”, whilst intrinsic and external regulation reflected the “why”. Based on a sample of 496 youth sports participants, structural equation modeling with a bootstrapping procedure was used to examine whether the indirect relationship between achievement goal orientation and self-esteem was conditional to motivational regulation. The results show partial support for the conditional process models. Specifically, task orientation was indirectly linked with self-esteem through competence need, and the relationship was stronger with higher levels of intrinsic regulation for sport. Furthermore, ego orientation was negatively associated with self-esteem through a positive relationship with competence frustration. However, this relationship emerged only for those higher in intrinsic regulation. External regulation did not emerge as a moderator, but presented a positive relationship with competence frustration. Findings are discussed in light of both Achievement Goal Theory and Self-Determination Theory, and underline the importance of considering both the “what” and “why” when attempting to understand motivation in youth sport.

## Introduction

Understanding motivation requires addressing both the direction of behavior; the “what”, and its energizing aspect; the “why” (Deci and Ryan, 1985). Thus, inspired by Vansteenkiste et al. (2014a), the purpose of the present study was to combine two prominent theories of motivation; namely Self-Determination Theory (SDT; Deci and Ryan, 2000) and Achievement Goal Theory (AGT; Nicholls, 1984), in order to investigate both aspects of motivation in the context of youth sports. The conceptual basis for the study included motivational regulations from SDT, reflecting “why” one is participating, and goal orientation from AGT to represent the “what” one is trying to achieve. Specifically, we asked whether the relationship between youth sports participants’ achievement goal orientation and self-esteem, through competence, is conditional upon motivational regulation.

Elliot and Thrash (2001) described the “why” as the energizing element of achievement behavior. We drew upon SDT as a theoretical basis for the “why”, which posits that motivation varies in the degree of self-determination. This can be seen on a continuum from extrinsic to intrinsic, along several distinct dimensions of motivation differing in quality depending on the underlying regulatory processes (Deci and Ryan, 2000). Extrinsic motivation consists of four different regulations. The first is external regulation, representing a highly controlled form of motivation, occurring when the source of motivation is alien to the person (e.g., being forced by a parent to participate in sports). Introjected regulation is also controlling, but the control is internal, often characterized by shame or guilt. Identified regulation is a more self-determined dimension, involving accepting and identifying with the underlying value of a given behavior. The final dimension of extrinsic motivation is integrated regulation, occurring when the value of a behavior is integrated within the self. Intrinsic regulation reflects complete self-determination, i.e., acting due to interest and enjoyment inherent in the activity itself, also in the absence of external prompts and rewards.

Previous investigations on the “what” and “why” of motivation have relied on a dichotomy of self-determined regulation (identified, integrated and intrinsic regulation) and controlled regulation (introjected and external regulation) (Vansteenkiste et al., 2014a). Self-determined regulation, compared to controlled, should lead to more facilitative outcomes through increased effort and persistence, less internal conflict, challenge appraisals, and protection from task-irrelevant temptations (Sheldon and Elliot, 1999; Deci and Ryan, 2000; Koestner, 2008; Ntoumanis et al., 2014). However, this method of combining qualitatively distinct regulations has been scrutinized as research suggests that considering the quality of motivation adds explanatory value even when accounting for the amount of self-determination (Howard et al., 2016). Thus, we employed intrinsic and external regulation, representing completely self-determined and non-self-determined regulation, to examine their unique contribution to outcomes.

Both AGT and SDT researchers have highlighted the importance of goal content when studying the implications of achievement behavior (Ryan et al., 1996; Elliot and Thrash, 2001). We elected to base the “what” on competence dimensions due to the achievement focus within sports. As AGT emphasizes the concept of competence, the theory offers a theoretically sound basis for the “what”. AGT is concerned with conceptions of competence, and posits a dichotomy in how it is construed (Nicholls, 1984). A task conception of competence is self-referenced, and ability is considered in regard to mastery, effort and learning. Conversely, an ego conception is other-referenced. Competence evaluation is based on normative standards. Further, a valid inference of ability requires exerting equal or less effort compared to others (Nicholls, 1984). Generally, the conceptions are thought to differentially relate to outcomes. Task orientation is linked with positive outcomes, whilst ego orientation relates to more adverse ones, particularly when perceived competence is low (Roberts, 2012). Vansteenkiste et al. (2014a) proposed the use of the hierarchical model of achievement goals, including avoidance and approach dimensions, to study the “what”. However, research has cast doubt over whether adolescents actually distinguish between approach and avoidance, and if they represent separate psychological realities (Roeser, 2004). Therefore, we employed the traditional dichotomous distinction of ego and task orientation.

Vansteenkiste et al. (2014a) acknowledged that the pursuit of a given goal can be differentially regulated, presenting important nuances in the consequences of its pursuit. Initial research offers support for this in the context of sports. For example, self-determined regulation of task-approach goals positively predicted game-specific pro-social behavior, enjoyment and performance satisfaction in volleyball players (Vansteenkiste et al., 2014b). Furthermore, controlling reasons underlying ego-approach goals have been linked with unfair functioning in competition, higher negative affect, and lower positive affect (Vansteenkiste et al., 2010). Conversely, self-determined regulation of ego-approach goals was associated with positive affect and subjective vitality (Vansteenkiste et al., 2010). Interestingly, a recent study on student athletes showed that goals and regulations interact to predict outcomes (Gaudreau and Braaten, 2016). Results showed stronger relationships between task-approach goals and goal attainment, and between ego goals and goal attainment, sport satisfaction, and positive affect for those with self-determined reasons. Moreover, both ego-approach and task-approach goals presented stronger relationships with negative affective states when high in controlled reasons.

Inspired by the aforementioned work, we wanted to investigate this combination using a contextual level of motivation for youth sports. The notion herein is that the “what” and “why” also exists in regard to sport participation in general. Specifically, participation in youth sports can be differentially regulated, reflecting important nuances in how the “what” will relate to outcomes. This approach is likely to have greater predictive value, as reasons for participation encompass more information compared to the reasons for specific goals. Eligibility criteria state that a moderator must precede the independent variable (Kraemer et al., 2008). As it is the energizing basis for achievement behavior (Elliot and Thrash, 2001), we placed motivational regulation as the moderator in our models. Furthermore, Vansteenkiste et al. (2014a) assessed the regulation, or reasons, underlying specific achievement goals. With this method, the “what” and “why” becomes inextricably linked with each other. However, we found it appropriate to measure regulation and orientation separately, adhering to the rule that the moderator and predictor should not be associated if one is to present a true conditional analysis (Kraemer et al., 2008). Thus, the aim of the current study was to investigate whether the underlying regulation of participation would moderate how achievement goal orientation related to outcomes.

A majority of the research investigating how the combination of the “what” and “why” relates to outcomes has neglected to offer an explanation on the mechanisms by which the influence operates through. According to SDT, it is in terms of basic psychological need satisfaction that the combination becomes meaningful (Deci and Ryan, 2000). Basic psychological needs are defined as innate psychological nutriments, fundamental to well-being (Deci and Ryan, 2000). SDT posits three separate needs; autonomy, competence and relatedness, respectively. Although universality is a feature of basic psychological needs, their relative salience can vary, for example by cultural factors dynamically contributing to their importance (Ryan and Deci, 2000). Competence is highly emphasized in sports, and perhaps the most pertinent in regard to self-perceptions (Kipp and Weiss, 2015). Furthermore, as goal orientations reflect the standards by which participants evaluate their competencies, the need for competence is very relevant. For this reason, competence was investigated solely, defined as an innate and appetitive desire to feel competent in one’s actions and interactions (Deci and Ryan, 2000). We also assessed competence need frustration, i.e., perceiving the need for competence actively obstructed, as it has independent relationships with antecedents and outcomes (Bartholomew et al., 2011).

A task orientation should lead to competence need satisfaction, as self-referenced standards have an internal locus of control. Conversely, ego orientation reflects a standard more dependent on aspects external to the self, making it more challenging to reach. Furthermore, the external locus of control may lead to competence frustration when faced with failure (Nicholls, 1984). However, we hypothesize that the “why” of participation presents important nuances in how the “what” relates to outcomes. Therefore, the relationship between goal orientation and competence should be considered in light of how the activity is regulated. Although need satisfaction has traditionally been seen as an antecedent of motivational regulation, recent longitudinal research suggests that regulations may in fact facilitate need satisfaction (Gunnell et al., 2014). As intrinsic regulation reflects a representation of an individual’s integrated sense of self, any activities regulated such are more connected to the need for competence, compared to externally regulated ones (Deci and Ryan, 1995). Additionally, as self-determined activity is afforded more effort, leading to activity absorption and better skill development, increases in actual competence are more likely (Sheldon and Elliot, 1999; Vansteenkiste et al., 2014a).

We aimed to move beyond only assessing the type or strength of goal orientation and regulation, by combining the two. Previous research has offered no support for an interaction of self-determined regulation and achievement goals on need satisfaction (Gillet et al., 2014; Delrue et al., 2016). However, moderation requires a great deal of power. Thus, by using less sophisticated analyses, i.e., multiple regression (versus structural equation modeling; SEM), and a lower number of participants, the ability to identify interactions may have been reduced in these studies (Hayes and Preacher, 2013). Furthermore, these investigations focused on the reasons underlying specific goals, not the regulation for participation in general. Therefore, extending previous research, we attempted to detect an interaction between goal orientation and motivational regulation when employing SEM with a larger sample and the regulations underlying participation *per se*.

Self-esteem is an evaluative component of self-perception, representing affective appraisals of one’s worth and importance (Fox, 2002). Unfortunately, puberty has been presented as a developmental marker associated with female athletes’ lowered self-perceptions (Monsma et al., 2006). As it is imperative to understand how self-esteem can be promoted in this period, we added self-esteem as an outcome in our model. Self-esteem is determined by specific concepts of competence (Marsh, 1986; Wagnsson et al., 2014), and recent work has shown competence to be the only basic psychological need to predict self-esteem (Kipp and Weiss, 2015). Furthermore, SDT posits that true self-esteem can only be facilitated through acting agentically and volitionally, and having one’s basic needs met (Deci and Ryan, 1995). Accordingly, goal orientations should contribute to self-esteem only to the extent that they are able to satisfy the need for competence. Therefore, we wanted to investigate if the relationship of goal orientation to competence is moderated by regulation, and if this extends to the indirect association from goal orientation to self-esteem. With this aim in mind, we deemed a conditional process analysis as the appropriate manner in which to test these relationships.

**FIGURE 1:**
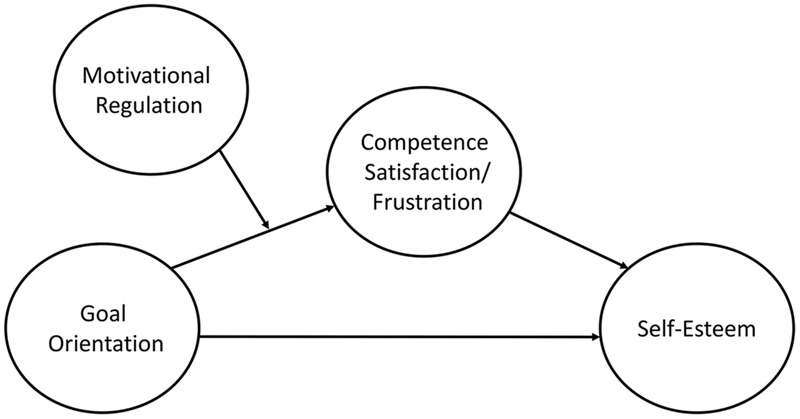
**The conceptual conditional process model**.

We tested several conditional process models (**Figure 1**), based on two different mediation sequences; *task goal orientation* – *competence satisfaction* – *self-esteem*, and *ego goal orientation* – *competence frustration* – *self-esteem*, respectively. We expected ego goal orientation to negatively relate to self-esteem through a positive relationship with competence frustration, and task goal orientation to positively relate to self-esteem through competence. We hypothesized that the relationship between task orientation and competence satisfaction would be stronger for those with high levels of intrinsic regulation, whilst the opposite was expected for the relationship between ego orientation and competence frustration. We also hypothesized that the relationship between task orientation and competence satisfaction to weaken with high levels of external regulation, whilst seeing a strengthening of the relationship between ego orientation and competence frustration.

## Materials and Methods

### Participants

Participants were 496 female soccer and handball players, ranging from 11 to 19 years of age (*M* age = 14.10, *SD* = 1.86). The Norwegian Centre for Research Data (NSD) approved the project prior to its commencement. The participants were recruited by contacting clubs directly. An information letter was sent to coaches, who upon accepting the invitation forwarded an information sheet to players and their parents/legal guardians. Parents or legal guardians as well as participants above the age of 18 were asked to indicate consent through a passive consent approach, which entailed giving the project leader a verbal or written refusal if they did not consent to participation. Participants were informed that participation was voluntary and consent could be withdrawn at any point. The data collection took place at the end of the season for soccer and midseason for handball, and the questionnaire was administered before or after a team training session, and took on average 20 min to complete.

### Measures

Participants responded to all items on a 5-point Likert-Scale, ranging from 1 (*strongly disagree*) to 5 (*strongly agree*). All scales were administered in Norwegian, following an extensive translation-back-translation procedure from English (Harkness, 1999).

*Achievement goal orientation* was measured based on work by Duda and Nicholls (1992), and items were preceded by the stem “I feel really successful in football/handball when…”. All items referred to the standards for feeling successful, and did not include validation concerns or choice components, thus more precisely reflecting competence standards. Six items measured *ego goal orientation* (e.g., “I’m the only one who can do the skill”), whilst seven items were used to assess *task goal orientation* (e.g., “I do my very best”). Previous research (e.g., White and Duda, 1994) has demonstrated acceptable psychometric properties for the use of this scale with youth sports participants.

The Behavioral Regulation in Sport Questionnaire (BRSQ) (Lonsdale et al., 2008) was used to measure *motivational regulation*. Participants were asked to rate how well the statements fit with their reasons for participating. Four items assessed *intrinsic regulation* (e.g., “Because I enjoy it”), and four items measured *external regulation* (e.g., “Because people push me to play”). Viladrich et al. (2013) offered support for the use of this scale with youth athletes in several European countries including Norway.

In regard to competence, the participants were asked about their general feelings and experiences on the team during the past month. *Competence need satisfaction* was assessed based on six items from the Intrinsic Motivation Inventory (IMI) (McAuley et al., 1989) (e.g., “I was pretty good”). McAuley et al. (1989) supported the psychometric properties of the scale in a sports context. C*ompetence need frustration* was measured with four items from the competence factor of the Psychological Need Thwarting Scale (PNTS) (Bartholomew et al., 2011) (e.g., “There were situations where I was made to feel useless”). Bartholomew et al. provided initial support for the reliability and validity of the scores attained from this measure.

Five items from the Short Version of the Self-Description Questionnaire (Marsh et al., 2010) were used to measure general *self-esteem*. The participants were asked how they generally felt in the past 3–4 weeks (e.g., “Overall, most things I did, I did well”). Previous research (e.g., Marsh et al., 2010; Papaioannou et al., 2013) has presented acceptable psychometric properties for the self-esteem items with youth athletes.

### Data Analyses

While most of the research to date has investigated conditional processes using regression analyses (Curran et al., 2013; Sardeshmukh and Vandenberg, 2016), we extended this work by employing SEM, with M*plus* 7.2 statistical software. To evaluate model fit, we relied on common goodness-of-fit indices, including comparative fit index (CFI), the root mean square error of approximation (RMSEA), and the standardized root mean square residual (SRMR). According to Little (2013), good fit is indicated by values close to or greater than CFI = 0.90, and less than 0.08 for RMSEA and SRMR, respectively.

As recommended by Hayes (2013), we first tested mediation, thereafter moderation, and subsequently all parameters were estimated simultaneously to test the moderated mediation. Interaction terms were created in M*plus* using the XWITH command. With this command, Mplus employs the latent moderated structural equations approach which offers unbiased, efficient estimates of interaction effects, robust toward departures from normality and non-linearity (Hayes and Preacher, 2013; Sardeshmukh and Vandenberg, 2016). An analysis of the index of moderated mediation was requested, which reflects the slope of the line representing the relationship between the moderator and the mediation link (Hayes, 2015). Estimates of the indirect effect were specified at low (-1SD), moderate (Mean), and high (++1SD) levels of the moderator. Furthermore, as these values are of an arbitrary nature, we also employed regions of significance, i.e., the Johnson–Neyman technique, by loop plotting the conditional indirect relationships in M*plus* (Hayes and Preacher, 2013; Muthén et al., 2016). This technique defines regions of moderator values at which the simple slope of the indirect relationship is significantly different from zero. All analyses were carried out with bias-corrected bootstrapping, with 5000 samples, reporting significance based on 95% bias-corrected confidence intervals for all effects.

The aforementioned fit indices are not applicable when running models with the XWITH interaction term in M*plus*. We therefore relied on the method presented by Sardeshmukh and Vandenberg (2016) to assess model fit. Baseline models were computed, where only main effects were specified for the moderator. Thereafter the Akaike Information Criterion (AIC) was compared between the baseline model and the model with the interaction term. A smaller AIC suggests less information loss, indicating a better fit to the data.

Interaction tests are low in statistical power (Hayes, 2015). Furthermore, SEM analyses with interactions rely on numerical integration and raw data, requiring great capacity for the computations. Combined with 5000 bootstrap samples, running simpler models was deemed more appropriate. Thus, we opted to analyze the achievement goal orientations separately, with two models for ego (ego/intrinsic and ego/external) and two models for task (task/intrinsic and task/external). This also favors parsimony, and attempts to reduce the likelihood of multicollinearity and potential type 2 errors. Furthermore, it facilitates interpretation, and is similar to that done in previous studies (Gaudreau and Braaten, 2016).

## Results

### Descriptive Statistics

Inspection of skewness and kurtosis revealed that all items met with the cut-off values of +/- 2 for skewness (George and Mallery, 2010). However, intrinsic regulation, external regulation, and task goal orientation presented numbers exceeding this for kurtosis. As suggested by Byrne (2012), we assessed changes in the *X^2^*-value when conducting confirmatory factor analysis (CFA) with both maximum likelihood (ML) and maximum likelihood estimation method with robust standard errors (MLR), for all three variables. The changes were substantial, suggesting non-normality. Based on this, the MLR estimator was applied, due to it being robust to non-normality (Muthén and Muthén, 1998-2012). All items loaded on their respective latent constructs (unstandardized estimates ranging from 0.67 to 1.24, all being statistically significant at *p* < 0.001). As Cronbach’s alphas are recognized as limited estimators of reliability, the latent variable model composite reliability, denoted by Rho (ρ), was computed to provide a less biased estimate (Raykov, 2009). Means, standard deviations, Rho and bivariate correlations are presented in **Table 1**. Correlations generally revealed an expected pattern between variables.

**Table 1:** Descriptive statistics, Rho, and bivariate correlations for latent variables.

	Raikov^1^	M *(SD)*	2	3	4	5	6	7
1 T–G–O	0.82 (0.79–0.85)	4.49 (0.51)	0.03	0.45^∗∗^	-0.22^∗∗^	0.61^∗∗^	-0.25^∗∗^	0.19^∗∗^
2 E–G–O	0.84 (0.82–0.86)	3.07 (0.90)		0.07	0.14^∗∗^	-0.09	0.21^∗∗^	0.01
3 C-Sat	0.92 (0.91–0.93)	3.60 (0.76)			-0.34^∗∗^	0.40^∗∗^	-0.17^∗∗^	0.37^∗∗^
4 C-Fru	0.79 (0.77–0.82)	2.34 (0.92)				-0.21^∗∗^	0.24^∗∗^	-0.44^∗∗^
5 I-Reg	0.78 (0.74–0.82)	4.69 (0.41)					-0.29^∗∗^	0.13^∗∗^
6 E-Reg	0.75 (0.70–0.80)	1.45 (0.59)						-0.17^∗∗^
7 SE	0.79 (0.76–0.83)	3.84 (0.70)						

*^∗∗^*p* ≤ 0.01; ^1^Confidence intervals for Rho in parentheses. T–G–O = task goal orientation, E–G–O = ego goal orientation, C-Sat = competence need satisfaction; C-Fru = competence need frustration, I-Reg = intrinsic regulation, E-Reg = external regulation, SE = self-esteem.*

### Confirmatory Factor Analyses

Initial CFA for ego orientation did not yield acceptable fit indices [(S–B χ^2^) = [df = 9, *N* = 495] = 69.377, *p* < 0.001; CFI = 0.93, RMSEA = 0.12[0.09–0.14] and SRMR = 0.05]. Modification indices (MI) revealed high residual covariance between item 5 and 6, respectively. Item phrasing indicated redundancy due to item overlap (Podsakoff et al., 2012); item 5 “I am the best player in my position”, and item 6 “I’m the best”. We therefore considered it acceptable to add a covariance link between the residual covariance associated with both items, as they relate to similar content. This resulted in excellent fit indices [(S–B χ^2^) = [df = 8, *N* = 495] = 27.656, *p* < 0.001; CFI = 0.98 and RMSEA = 0.07[0.04–0.09], and SRMR = 0.03].

The initial CFA for self-esteem showed non-acceptable fit indices [(S–B χ^2^) = [df = 5, *N* = 488] = 47.877, *p* < 0.000; CFI = 0.92, RMSEA = 0.13[0.10–0.17], and SRMR = 0.04]. Again, MI revealed high residual covariance, between item 2 and 4, respectively. Both items were negatively phrased, and thereafter turned in SPSS. Item 2 stated “I was worthless” and item 4 stated “Little of what I did turned out well”. Adding a covariance link between item 2 and 4 yielded excellent fit indices [(S–B χ^2^) = [df = 4, *N* = 488] = 10.669, *p* < 0.05; CFI = 0.99, RMSEA = 0.06[0.00–0.06] and SRMR = 0.02]. This is consistent with the approach employed by Papaioannou et al. (2013) when examining the factor structure of the scale across five European countries, including Norway. The remaining CFAs for task goal orientation, competence satisfaction, competence frustration, external regulation and intrinsic motivation yielded acceptable fit indices.

### Mediation

Results revealed a significant sequence for task orientation – competence need – self-esteem [(S–B χ^2^) = [df = 131, *N* = 496] = 176.817, *p* = 0.00; CFI = 0.99, RMSEA = 0.03[0.02–0.04], and SRMR = 0.04]. Specifically, a significant total positive effect of task goal orientation on self-esteem was observed (β = 0.24, 95% CI_BC_:0.12,0.35), which included a positive indirect path (β = 0.22, 95% CI_BC_:0.14,0.31). The direct path between task goal and self-esteem was non-significant.

The ego goal orientation – competence frustration – self-esteem model showed acceptable fit indices [(S- B χ^2^) = [df = 85, *N* = 496] = 233.599, *p* = 0.00; CFI = 0.94, RMSEA = 0.06[0.05–0.07] and SRMR = 0.06]. The total effect of ego orientation on self-esteem was non-significant. However, an indirect negative path via competence frustration emerged (β = –0.05, 95% CI_BC_:–0.11,0.06), whilst the direct link was non-significant. According to Hayes (2013), neither the direct or total effect must be significant to support mediation. Thus, as the present results supported the hypothesized indirect relationships, we were confident in conducting further analyses. However, the direct paths were omitted from the conditional process models.

### Moderation

**FIGURE 2:**
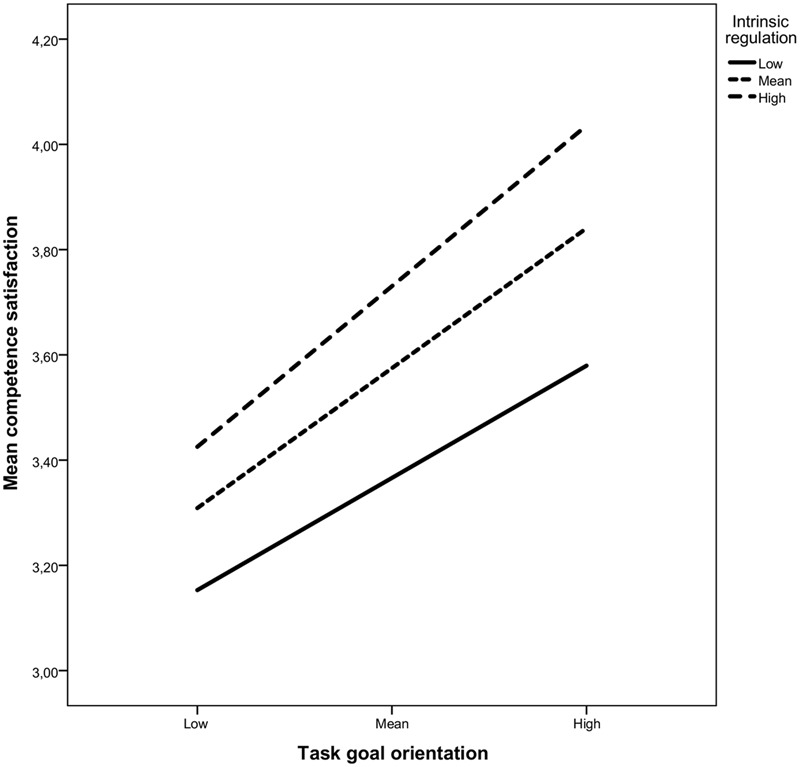
**Moderating role of intrinsic regulation on the relationship between task goal orientation and competence need satisfaction**.

Conditional effects for both task orientation (*B* = 0.67, 95% CI_BC_:0.45,0.93) and intrinsic regulation (*B* = 0.19, 95% CI_BC_:0.08,0.29) were significant, as was the interaction term (*B* = 0.15, 95% CI_BC_:0.04,0.26) (**Table 2**). Simple slopes analyses, presented in **Figure 2**, showed that the association between task orientation and competence need were significant at all levels, but increased in strength from low (–1SD; *B* = 0.61, CI_BC_:0.39,0.88), to moderate (Mean; *B* = 0.67, CI_BC_:0.45,0.93), to high levels of intrinsic regulation (+1SD; B = 0.73, 95% CI_BC_:0.50,1.00). Further, the interaction term in the task goal/external model was non-significant.

**Table 2:** Simple moderation for task goal orientation models.

	Unstandardized coefficients (95% CI_BC_)			
	Task	Intrinsic	External	Interaction
Competence	0.67 (0.45,0.93)^∗∗^	0.19 (0.08,0.29)^∗∗^	–	0.15 (0.04,0.26)^∗^
Competence	0.76 (0.58,0.90)^∗^	–	-0.06 n.s.	-0.08 n.s.

*^∗^*p* < 0.05, ^∗∗^*p* < 0.001, n.s. = non-significant.*

**FIGURE 3:**
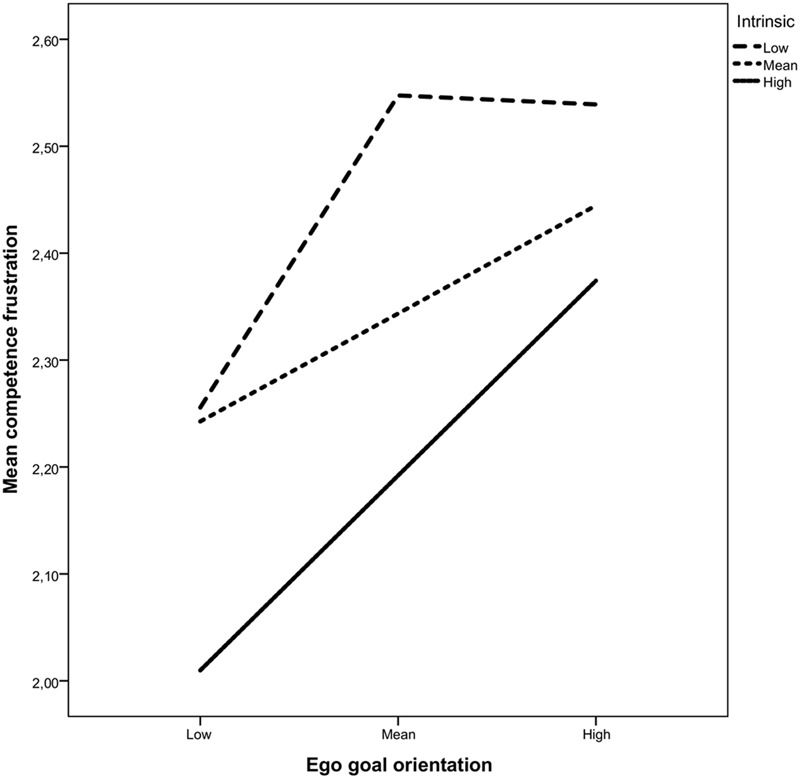
**Moderating role of intrinsic regulation on the relationship between ego goal orientation and competence frustration**.

Only the link from intrinsic regulation to competence frustration was significant in the ego/intrinsic model (B = –0.28, 95% CI_BC_:–0.40,–0.18), yet a significant interaction term emerged (*B* = 0.19, 95% CI_BC_:0.06,0.31) (**Table 3**). Simple slopes analyses (**Figure 3**), showed that the path between ego orientation and competence frustration was only significant at high levels of intrinsic regulation (+1SD; *B* = 0.27, 95%CI_BC_:0.08,0.47). In the ego/external regulation model, only external regulation presented a significant relationship with competence frustration (*B* = 0.31, 95%CI_BC_:0.20,0.43), whilst the interaction term was non-significant.

**Table 3:** Simple moderation for ego goal orientation models.

	Unstandardized coefficients (95% CI_BC_)			
	Ego	Intrinsic	External	Interaction
Competence-Frustration	0.03n.s.		0.31 (0.20,0.43)^∗∗^	-0.12 n.s.
Competence-Frustration	0.09 n.s.	-0.29 (–0.38,–0.20)^∗^	-	0.19 (0.08,0.31)^∗^

*^∗^*p* < 0.05, ^∗∗^*p* < 0.01, n.s. = non-significant.*

### Moderated Mediation

The moderated mediation index for intrinsic regulation on the association between task orientation and self-esteem was significant (*B* = 0.06, 95% CI_BC_:0.02,0.12). A conditional indirect effect of task goal on self-esteem through competence need emerged, significant at low (–1SD; *B* = 0.22, 95% CI_BC_:0.11,0.39), moderate (Mean; *B* = 0.29, 95% CI_BC_:0.16,0.47), and high levels of intrinsic regulation (+1SD; *B* = 0.35, 95% CI_BC_:0.20,0.56). This was supported by the loop plot results, showing that the conditional indirect relationship was significant at all levels of intrinsic regulation, as such the regions of significance was the entire samples range of intrinsic regulation (range = 2.5–5). Furthermore, the slope was positive, showing an increase in the strength of the indirect effect with increasing levels of intrinsic regulation. The association between task orientation and self-esteem therefore appears not to be conditional upon intrinsic regulation, but the strength of the association is.

The interaction term for task goal/external regulation was non-significant. However, this does not reflect a quantification of the relationship between the moderator and the indirect effect and therefore one cannot infer that the indirect effect is not conditional upon the moderator (Hayes, 2015). Thus, moderated mediation analyses were conducted, revealing a non-significant index of moderated mediation (*B* = –0.03, n.s.). Accordingly, it appears that the relationship between task orientation and self-esteem through competence satisfaction was not conditional to external regulation.

The moderated mediation index for intrinsic regulation on the relationship between ego orientation and competence frustration was significant (*B* = –0.07, 95% CI_BC_:–0.13,–0.03). Simple slopes, depicted in **Table 5**, revealed a conditional indirect effect of ego orientation on self-esteem, through competence frustration but only at high levels of intrinsic regulation (+1SD; *B* = –0.06, 95% CI_BC_:–0.12,–0.01). The regions of significance test showed that the conditional indirect relationship was significant when intrinsic regulation was equal to, or higher than, 4.87 (*B* = –0.05, 95% CI_BC_:–0.10,–0.01). Although this is a high number, on a scale of 5, it does reflect the responses of 44.15% of the sample. As such, there is enough data within this region of significance to offer a reliable finding. The results therefore suggests that the negative association between ego goal orientation and self-esteem, through increased competence frustration, is conditional upon high levels of intrinsic regulation.

Similarly, to the task model, external regulation showed no interaction with ego orientation in the simple moderation. Furthermore, the moderated mediation index was non-significant (*B* = 0.04, n.s.), indicating that external regulation did not moderate the relationship between ego orientation and competence frustration.

### Baseline Models

Results, depicted in **Tables 4, 5**, showed that only the task goal/external regulation model presented a larger AIC when including the interaction term. This indicates that the presence of the interaction is favored in the task/intrinsic, ego/intrinsic, and ego/external models, statistically speaking (Sardeshmukh and Vandenberg, 2016).

**Table 4:** Conditional indirect effects models with task goal orientation.

Moderator value (intrinsic regulation)	Conditional indirect effect of task goal orientation on self-esteem at mean and ±1SD levels of intrinsic regulation					
	Bootstrap indirect effect	Boot SE	95%L CI_BC_	95%U CI_BC_	Baseline AIC	Interaction AIC
Index of mod-med	0.06	0.03	0.02	0.12	19948.454	19942.036
–1SD intrinsic	0.22	0.08	0.11	0.39		
Mean intrinsic	0.29	0.08	0.16	0.47		
+1SD intrinsic	0.35	0.09	0.20	0.56		

**Moderator value (external regulation)**	**Conditional indirect effect of task goal orientation on self-esteem at mean and ±1SD levels of external regulation**					
	**Bootstrap indirect effect**	**Boot SE**	**95%L CI_BC_**	**95%U CI_BC_**	**Baseline AIC**	**Interaction AIC**

Index of mod-med	-0.03	0.03	n.s.	n.s.	21938.905	21939.405

*Bootstrap *N* = 5000. Unstandardized coefficients are depicted. 95%L CI_*BC*_ = 95% confidence interval lower limit. 95%U CI_*BC*_ = 95% confidence interval upper limit. Bias corrected confidence intervals are reported. AIC = Akaike Information Criterion.*

**Table 5:** Conditional indirect effects models with ego goal orientation.

Moderator value (intrinsic regulation)	Conditional indirect effect of ego goal orientation on self-esteem at mean and ±1 SD levels of intrinsic regulation					
	Bootstrap indirect effect	Boot SE	95%L CI_BC_	95%U CI_BC_	Baseline AIC	Interaction AIC
Index of mod-med	-0.07	0.03	-0.13	-0.03	21763.384	21756.935
–1SD intrinsic	-0.01	0.03	n.s.	n.s.		
Mean intrinsic	-0.03	0.03	n.s.	n.s.		
+1SD intrinsic	-0.06	0.03	-0.12	-0.01		

**Moderator value (external regulation)**	**Conditional indirect effect of ego goal orientation on self-esteem at mean and ±1 SD levels of external regulation**					
	**Bootstrap indirect effect**	**Boot SE**	**95%L CI_BC_**	**95%U CI_BC_**	**Baseline AIC**	**Interaction AIC**

Index of mod-med	0.04	0.03	n.s.	n.s.	23591.448	23590.311

*Bootstrap *N* = 5000. Unstandardized coefficients are depicted. 95%L CI_*BC*_ = 95% confidence interval lower limit. 95%U CI_*BC*_ = 95% confidence interval upper limit. Bias corrected confidence intervals are reported. AIC = Akaike Information Criterion.*

## Discussion

The present study examined several conditional process models in which the association between achievement goal orientation and self-esteem functioned through competence need satisfaction or frustration, conditional upon the levels of intrinsic or external regulation for sport. The results offered partial support for the hypothesized conditional relationships. Specifically, intrinsic regulation appeared to moderate the relationship between task orientation and competence need, and the relationship between ego orientation and competence frustration, respectively.

### Task Goal Orientation Models

Self-esteem has been shown as an outcome of more specific concepts of competence (Marsh, 1986; Wagnsson et al., 2014; Kipp and Weiss, 2015). Consistent with this, the simple mediation analysis indicated that task goal orientation was related to self-esteem, completely through competence satisfaction. First, this supports the notion that competence satisfaction is readily facilitated with a task orientation, potentially due to a more internal locus of control making the standard more attainable (Rotter, 1966). Second, it suggests that the need for competence in youth sport contributes to a general positive sense of self. According to the psychological centrality hypothesis, the participants appear to value sport-specific competence, which is why it contributes to their general self-esteem (Marsh, 1986). However, the result is not consistent with previous research reporting a direct association between task orientation and self-esteem (Kavussanu and Harnisch, 2000). The equivocal findings may be explained by how competence is measured. Kavussanu and Harnisch (2000) relied on normative-based perceptions of ability. In light of this, their findings seems logical, as the self-perceptions of someone high in task orientation should not, at least not fully, depend on normative standings. However, the present study shows that when the participants report competence level according to how they define it themselves, the relationship between task orientation and self-esteem operated completely through the need for competence.

Contrary to previous work, the present results showed an interaction of task orientation and regulation on the need for competence (Gillet et al., 2014; Delrue et al., 2016). Indeed, conditional process analyses indicated that the strength of the indirect link between task orientation and self-esteem was conditional on the level of intrinsic regulation. More specifically, whilst the positive indirect effect was significant at all reported levels of intrinsic regulation, the association was stronger with increasing levels of intrinsic motivation. Thus, how strongly task orientation in sports is related to general self-esteem through competence is conditional to the degree that participation is regulated intrinsically. This is consistent with SDT, suggesting that self-esteem is facilitated through acting agentically and volitionally (Deci and Ryan, 1995). A possible explanation is that intrinsically regulated participation is likely to spur sustained effort over time, leading to activity absorption and greater skill development (Sheldon and Elliot, 1999; Koestner, 2008; Vansteenkiste et al., 2014a). This is consistent with previous findings in sports, as the interaction of autonomous reasons and high levels of task-approach goal has been associated with higher perceptions of self-reported goal attainment (Gaudreau and Braaten, 2016).

The indirect association between task orientation and self-esteem was not conditional to the level of external regulation. Furthermore, no main effects of external regulation were found, and comparison to the baseline model did not offer support for the interaction. These findings may in part be due to the low levels of external regulation reported by the participants, suggesting that this is not a big part of their motivation for sport. Nevertheless, the result does corroborate previous research, reporting that controlled motives for goal pursuit did not relate to positive outcomes such as need satisfaction and effort (Delrue et al., 2016). Indeed, it has been suggested that external regulation may primarily relate to need frustration and not need satisfaction (Deci and Ryan, 2000; Delrue et al., 2016).

### Ego Goal Orientation Models

Ego orientation emerged as negatively associated with self-esteem through competence frustration. This extends previous research by showing that self-esteem is related to the frustration of specific concepts of competence (Marsh, 1986; Kipp and Weiss, 2015). Furthermore, consistent with the assumptions of AGT (Nicholls, 1984), the results suggest that high levels of ego orientation are likely to contribute to feelings of competence need frustration. The explanation for this relationship may lie in the nature of an ego orientation, and the increased challenge associated with the other-referenced criteria for success. First, the increased difficulty reduces the likelihood of meeting the criteria for success. Second, the normative nature of the criteria means that attainment is dependent on external factors (e.g., the performance of others, the opportunity to compete and competitive conditions). Therefore, not only is failure more likely, the failure itself is prone to be attributed externally (Nicholls, 1984). Competence frustration is defined as perceiving the need for competence actively obstructed (Bartholomew et al., 2011). Thus, if failure is attributed to external factors, these factors are likely to be perceived as actively obstructing the pursuit of success. This will be experienced as competence frustration rather than a lack of competence.

The conditional process analysis showed that the indirect relationship between ego orientation and self-esteem was conditional on the level of intrinsic regulation. Interestingly, the results of the Johnson-Neyman technique showed that the negative relationship between ego orientation and self-esteem was apparent for those with the highest level of intrinsic regulation, specifically a level of 4.87 or higher. Somewhat counter to what we would expect, this warrants further discussion, and the findings are threefold. First, those low or moderate in intrinsic regulation were higher in competence frustration at all levels of ego orientation, compared to those high in intrinsic regulation. This points to the implications of being lower in intrinsic regulation for feelings of competence, regardless of level of ego orientation. This was supported by a negative main effect of intrinsic regulation to competence frustration. Second, at moderate and low levels of intrinsic regulation, increases in ego orientation were not significantly influential in terms of competence frustration, and subsequent self-esteem. Intrinsic regulation is a representation of, and emanating from, an individual’s integrated sense of self, and is closely connected to psychological needs (Deci and Ryan, 1995; Deci and Ryan, 2000). Thus, goal orientation is more likely to be meaningful to someone who is highly intrinsically regulated. Indeed, according to Deci and Ryan (1995), for something to contribute to true self-esteem, is must be reflective of such an integrated sense of self. It follows therefore that lower levels of intrinsic regulation may indicate that the activity is not representing the self, and an ego orientation may not have the power to influence competence and self-esteem.

The third point of discussion is that higher levels of ego orientation were associated with higher levels of competence frustration under conditions of high intrinsic regulation. Thus, it seems that *what* type of competence you are striving for in an intrinsically regulated activity matters. This is not consistent with SDT, which posits that if an activity represents the values and interest of the inner self, the achievement process will lead to positive outcomes (Sheldon and Elliot, 1999). However, even if intrinsic regulation is inherently positive, it cannot affect the objective aspects that make the standards of success that accompany an ego orientation more challenging. The aforementioned effort, activity absorption and skill development that intrinsic regulation promotes (Sheldon and Elliot, 1999; Deci and Ryan, 2000; Koestner, 2008) will only matter for ego-oriented individuals if it equates to normative performance. Additionally, high levels of effort in combination with failure is perhaps the most detrimental event, in terms of perceived competence, for those high in ego orientation (Nicholls, 1984).

Results suggest that the quality of regulation alone may not be sufficient to ensure positive outcomes. Furthermore, it appears that intrinsic regulation may even increase sensitivity toward less facilitative definitions of competence (i.e., ego orientation), due to the increased importance that the activity holds for the person (Deci and Ryan, 2000). However, the relatively high mean score for intrinsic regulation suggests that the majority of the participants seemed to be self-determined in their engagement. This means that comparisons between levels must be interpreted with caution. Accordingly, further research is needed to see if the results can be replicated, particularly in context where regulation is less likely to be so positively skewed.

Similar to the task/extrinsic model, the relationship between ego orientation and self-esteem was not conditional to the level of external regulation. Again, this could be in part due to the low levels of external regulation reported. Nevertheless, main effects for external regulation showed a positive relationship with competence need frustration, suggesting that external regulation operates independently to predict competence frustration, regardless of the level of ego goal orientation. Furthermore, this supports previous findings suggesting that external regulation relates primarily to need frustration and not need satisfaction (Deci and Ryan, 2000; Delrue et al., 2016).

### Limitations, Strengths, and Conclusion

The current study is not without limitations. First, the very goal of moderation and mediation analyses is to detect possible causal processes (Hayes and Preacher, 2013). The cross-sectional design of the present study is therefore a limitation, as no causality inferences can be made. Second, the sample included only female team sports athletes, which limits generalizability. Additionally, although the relatively large age span can be seen as a strong point, we do not know whether it affected the results through differences in understanding of the aspects measured. Third, the present study measured goal orientation to represent the standards by which the participants judge their competencies, with an assumption that competence demonstration is of importance. However, there may be several other salient aims, such as social ones (Urdan and Maehr, 1995). Last, the measure of competence need satisfaction employed herein is reflective of perceived competence. We acknowledge that, theoretically, one can be satisfied in terms of competence without being high in perceived competence. As such, our measurement may not appropriately capture the complexity of competence need satisfaction, and results should be interpreted with this in mind.

Notwithstanding the aforementioned limitations, the present study has several strengths. First, the use of SEM is a strong point (Sardeshmukh and Vandenberg, 2016). One of the principal benefits of using SEM is the ability to correct for the attenuating effects of measurement error by using latent variables (Hayes and Preacher, 2013). This may have allowed us to identify previously undetected relationships. Also, a conditional process analysis is an appropriate manner in which to assess the combination of the “what” and “why” of motivation. Here the large sample size is particularly pertinent. Furthermore, using need satisfaction and frustration to explain the mechanism by which the combination of the “what” and “why” contributes to outcomes appears theoretically attractive. Finally, to our knowledge, this is the first study to investigate this conditional process with youth sports participants.

In sum, this study demonstrates that task goal orientation is associated with general self-esteem, through the facilitation of competence, and the relationship appears to be stronger with higher levels of intrinsic regulation. Conversely, ego goal orientation seems to oppose self-esteem levels, by contributing to competence frustration, and being highly intrinsically regulated for the activity may not aid against it. Further, the results have practical implications for coaches and parents, particularly pointing to the importance of considering both the “what” and “why” of participation when attempting to optimize self-perceptions. For example, participants who are highly intrinsically regulated may be at risk of reduced self-esteem if they are highly ego oriented. As such, it is important to promote *both* intrinsically regulated activity and a task goal oriented view of competence. A recently devised training program for coaches, entitled the Empowering Coaching^TM^, is based on postulates of both SDT and AGT, and may prove fruitful in facilitating both (Duda, 2013). Lastly, future research should seek to replicate these findings, employing longitudinal data. The results of such investigations can improve our understanding of athletes’ participation in sports and thereby help us make it more psychologically beneficial.

## Ethics Statement

This study was carried out in accordance with the recommendations of the Norwegian Centre for Research Data (NSD) with informed consent from all subjects. The ethical review did not require us to obtain written consent, due to the complete anonymity of the responses and lack of sensitive health data collected. Thus, and in accordance with the guideline put forward by the NSD, we employed a passive consent form requiring participants and parents/legal guardians to give the project leader verbal or written refusal if they did not consent to participation. The study was carried out in accordance with the Declaration of Helsinki, and the protocol was approved by the NSD.

## Author Contributions

SG is the first author, and has been involved in all aspects of the study, from the conception of the work, data collection, data analyses, interpretation of the data, and drafting and revising the manuscript. PA has contributed to the design, acquisition and interpretation of the data. He has also contributed to drafting and revising the manuscript. YO has contributed to the conception of the study, as well as the acquisition and interpretation of the data. He has also engaged in drafting the work and revising it. All three authors have given final approval for submission, and are in agreement regarding the accountability of the work.

## Conflict of Interest Statement

The authors declare that the research was conducted in the absence of any commercial or financial relationships that could be construed as a potential conflict of interest.
